# An Investigation into the Vancomycin Concentration in the Cerebrospinal Fluid Due to Vancomycin Intraventricular Administration in Newborns: A Study of 13 Cases

**DOI:** 10.1097/MD.0000000000000922

**Published:** 2015-06-05

**Authors:** Nobuaki Matsunaga, Ken Hisata, Toshiaki Shimizu

**Affiliations:** From the Faculty of Medicine, Department of Pediatrics, Juntendo University, Tokyo, Japan.

## Abstract

Treatment against shunt infection by transvenous antimicrobial treatment is difficult, with a high risk of relapse. Consequently, to maintain a sufficient cerebrospinal fluid (CSF) concentration, intraventricular administration is utilized in combination with the transvenous administration of vancomycin (VCM). Few studies have so far investigated the optimum administration dose for newborns and the concentration in the CSF. Therefore, we chronologically measured the VCM concentration in the CSF after VCM intraventricular administration in newborns and attempted to elucidate the optimum administration method.

The participants consisted of newborns admitted to Juntendo University Neonatal intensive care unit from March 2007 to June 2011 who underwent interventricular shunting placement. VCM was intraventricularly administered to 10 patients for a total of 13 cases. The CSF concentration of VCM was chronologically measured at 12 to 120 hours following the intraventricular administration of VCM.

The intraventricular administration groups with VCM of 20 (n = 6) and 10 mg (n = 2) had a high concentration in the CSF at 24 hours following administration (95–168 mg/L), with the concentration remaining high at 72 hours (13.2–72 mg/L). At the same time, in the 5 mg group (n = 5), the concentration in the CSF 24 hours following VCM administration was sufficiently maintained (33.2–62.9 mg/L), with a sufficient trough concentration still maintained at 72 hours (11.7–16.5 mg/L).

The concentration in the CSF is prolonged in newborns, thus allowing a sufficient therapeutic range to be maintained even at an intraventricular administration of 5 mg. It is therefore believed that the monitoring of the CSF is very important regarding the administration interval because the VCM concentration in the CSF differs depending on the case.

## INTRODUCTION

An interventricular shunt is a device inserted with the objective of applying intracranial pressure against the increase in intracranial pressure due to hydrocephalus, intracranial bleeding, brain tumors, etc. Shunt infection due to device insertion is 1 major complication following surgery, with hydrocephalus related to shunt infection becoming a major risk factor in the subsequent developmental prognosis. Even when sufficient measures against infections and careful shunt insertion are carried out, the incidence of shunt infection is still high, namely, ranging from 8% to 12%.^1–3^

In fact, intravenous antimicrobial administration before and following surgery is normally performed with the objective of preventing the occurrence of shunt infection; however, the effect of such treatments remains limited.^4^

Early diagnosis, appropriate antimicrobial treatment, and removal of the infected device improve the prognosis of shunt infection.^1,5^ However, the relapse rate following treatment is high, ranging from 22% to 24%.^6,7^ Therefore, a variety of investigations have been carried out to suppress relapse. Some studies mention that the relapse rate declines upon secondary treatment involving the reinsertion of the shunt after confirming the negative conversion of bacteria by external ventricular drainage, rather than temporarily replacing the infected shunt.^8,9^

In many cases, the cause of relapse is incomplete treatment resulting from the suppression of the transfer of drugs to the cerebrospinal fluid (CSF) due to the blood-brain barrier. Moreover, biofilm formation in the shunt may also be another possible cause. In fact, the transfer rate to the CSF by transvenous antimicrobial administration has been reported to range from 20% to 30% in the acute phase, even regarding vancomycin (VCM). However, when inflammation improves as a result of treatment, transition to the cardiac ventricle further declines, thus rendering sufficient maintenance of the drug concentration in the CSF difficult despite transvenous antimicrobial administration according to the degree of meningitis treatment.^10^

Moreover, another method that is sometimes performed involves eliminating bacteria by directly administering VCM into the cerebral ventricle, and thereby maintaining the level of drug concentration in the cerebral ventricle.^11–14^

The literature investigating the drug concentration in the CSF upon intraventricular administration of VCM is limited. In adults, the intraventricular administration of 20 mg is typical. Although administering from 10 to 20 mg at 24-hour intervals is suggested regarding infants, in recent years, some studies mention that a dose of 5 mg at 24-hour intervals is sufficient.^15^ In children, a high VCM concentration in the CSF level tends to be prolonged. As a result, the administration of 5 mg has been reported to be sufficient for intraventricular VCM administration, taking into account the amount of CSF disposal.^15^ Regarding the optimal dosage, they also mentioned that monitoring the intraventricular VCM concentration is useful because the VCM concentration in the CSF varies depending on the case. However, few investigations into such administration in children exist, with the data further limited regarding newborns.^15^

Our objective in this study was, therefore, to determine the appropriate intraventricular administration dose along with the interval of VCM for intraventricular VCM administration for shunt infections in newborns.

## PARTICIPANTS AND METHODS

A retrospective investigation was carried out based on the medical records of 10 patients and 13 cases that underwent ventricular shunt placement (Ommaya tube/ventriculoperitoneal shunt) at the Department of Neurosurgery upon admission to neonatal intensive care unit of Juntendo Hospital in the 5 years from March 2007 to June 2011.

The inclusion criteria were patients undergoing the intraventricular administration of VCM following surgery, in which the measurement of the CSF concentration of VCM was possible. The intraventricular administration dose of VCM was determined by the attending physician. The CSF was sampled from 12 to 120 hours following the last intraventricular VCM administration when absorbing CSF with the objective of alleviating intracranial pressure. The final 2 mL of extracted CSF was used to measure the VCM concentration in the CSF. The CSF was immediately centrifuged for 5 minutes at 3000 revolutions to eliminate the precipitate, then measured by latex agglutination inhibition. Furthermore, regarding the intraventricular administration of VCM, 1 g of VCM was dissolved in 200 mL saline and the required amount was then administered.

Ethical approval was not necessary and the requirement of written informed consent was waived because of the retrospective nature of the study.

## RESULTS

The patient characteristics are shown in Table 1. The mean gestational age (standard deviation) was 34-week 5 day (±5week 3day), mean birth body weight was 2213 g (±923), and sex was 8 of 13 male cases. The underlying diseases were as follows: 5 cases of myelomeningocele; 3 cases of hydrocephalus following bleeding; 2 cases of congenital hydrocephalus; 2 cases of encephalocele; and 1 case of brain tumor. The cells in CSF were elevated in 10 cases and the glucose level decreased (<40 mg/dL) in 11 cases. Pathogenic bacteria were detected in the CSF in 6 cases.

**TABLE 1:**
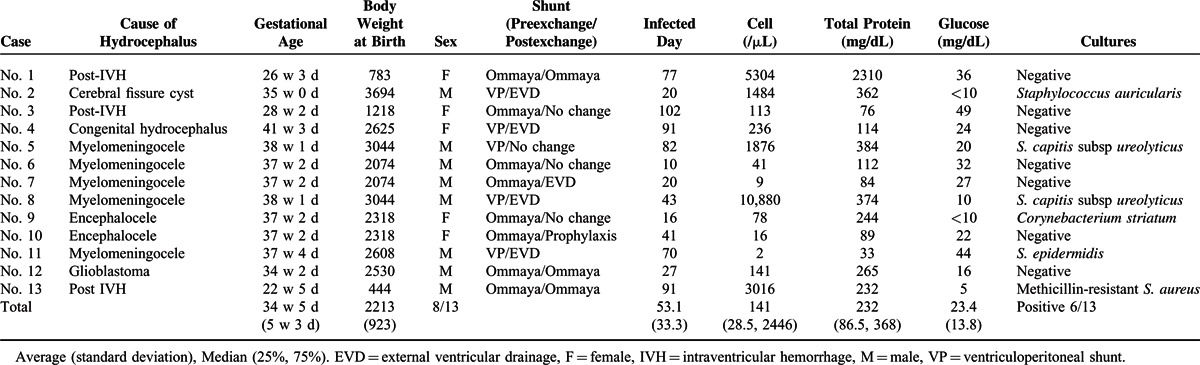
Clinical Characteristics of 13 Cases With Undergoing the Intraventricular Administration of Vancomycin

The patient treatments and prognosis are shown in Table 2. The mean infected day was 53.1 (±33.3), mean body weight at infected day was 3007 g (±1241). Intravenous administration was concomitantly utilized in 8 of 13 cases, with an average administration of 40.5 (±9.2) mg/kg/day when they were evaluated at discharge. The auditory brain stem response (ABR) was normal in 9 cases, whereas excluding 1 case observed with abnormalities in the ABR before administration. No cases were observed with any convulsions during the course. There were 2 cases demonstrating a relapse after being administered 10 and 20 mg, respectively.

**TABLE 2:**
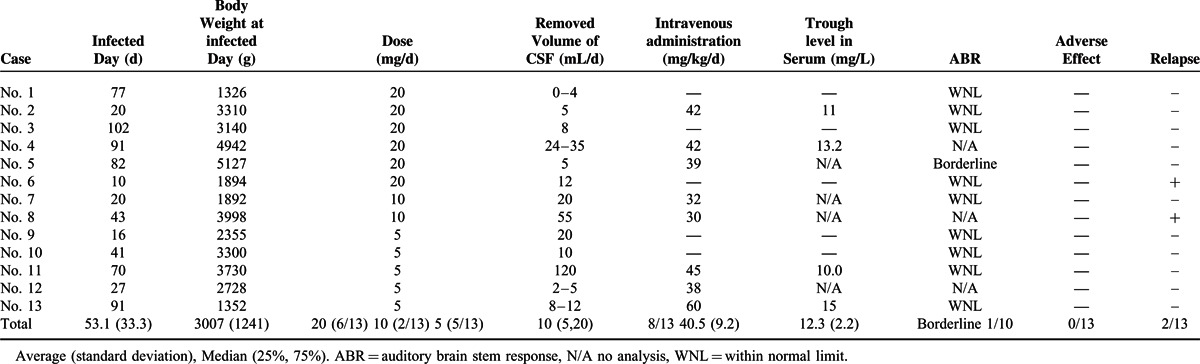
Patient Treatments and Prognosis

**FIGURE 1:**
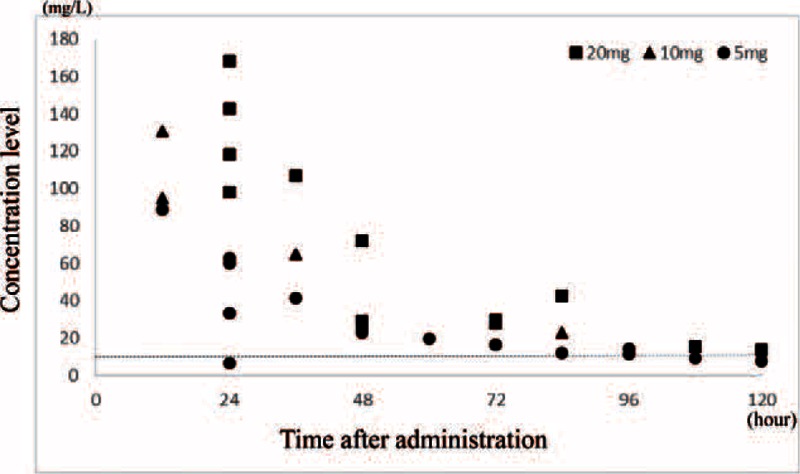
The monitoring of the vancomycin concentration in the cerebrospinal fluid. It was found that the cerebrospinal fluid concentration may still be sufficiently maintained 72 hours following administration. Dotted line = the recommended trough level (10 mg/L).

The intraventricular administration of 20 mg was carried out in 6 cases (Nos. 1–6) (Table 3, Figure 1). The average VCM concentration in the CSF at 24 hours following administration was 125.0 mg/L (±30.1), thus exhibiting a very high concentration. Moreover, the VCM concentration in the CSF was still prolonged at 28 (No. 5) and 29.5 mg/L (No. 6) at 72 hours following administration.

**TABLE 3:**
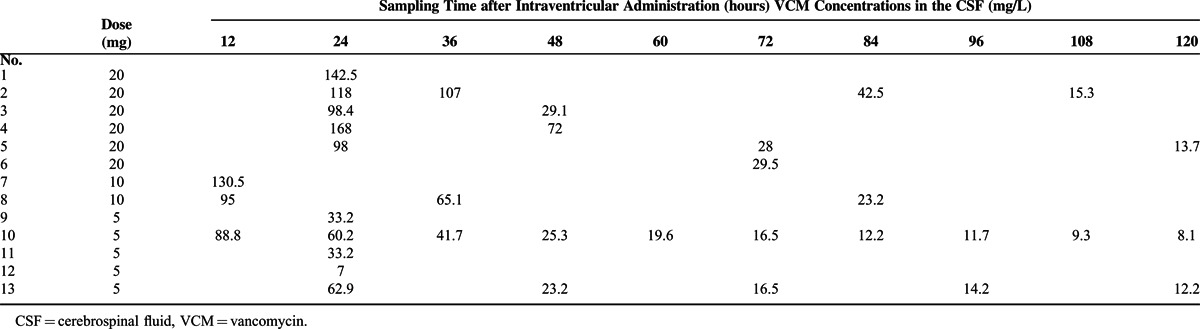
The Vancomycin Concentrations in the Cerebrospinal Fluid

The intraventricular administration of 10 mg was carried out in 2 cases (Nos. 7 and 8) (Table 3, Figure 1). The VCM concentration in the CSF at 12 hours following administration was high, namely, 95 mg/L for No. 8 and 130.5 mg/L for No. 7. Moreover, the VCM concentration in the CSF was still prolonged at 23.2 mg/L for No. 8 at 84 hours following administration.

The intraventricular administration of 5 mg was carried out in 5 cases (Nos. 9–13) (Table 3, Figure 1). Excluding 1 case (No. 11) with an excessive disposal of CSF (120 mL/day), the average VCM concentration in the CSF at 24 hours following administration was 39.3 (±22.9) mg/L, and the intraventricular VCM concentration was sufficiently maintained. Moreover, at 72 hours following administration, the VCM concentration in the CSF was still maintained at 16.5 mg/L (Nos. 10 and 13).

## DISCUSSION

The CSF concentration varied regarding the intraventricular administration of VCM in newborns in our hospital, at an administration of 20 mg, it was confirmed that the concentration in the CSF is prolonged at a high level. The result was the same regarding the administration of 10 mg. It is believed that the recommended trough level of CSF against S*taphylococcus aureus, S. epidermidis*, which is the major pathogenic bacteria of shunt infection, is 5 to 10 mg/L.^16^ Moreover, even in the bacteriological investigation using CSFs, the time kill curve test of VCM in S*. aureus, S. epidermidis* at ≥10 mg/L reveals that the effect thereof does not change.^17^ At the same time, the possibility of adverse effects due to an overdose is of concern along with aftereffects such as auditory disorders and central nervous system disorders. At present, the intoxication region of the established concentration in the CSF and the critical region of adverse effects are unknown.^5,18–20^ Conversely, some studies mention that potential auditory disorders and central nervous system disorders decline by making the CSF concentration of VCM ≤20 mg/L.^21^ In our investigation, excluding the 1 case observed with abnormal findings before administration, ABR was normal in all cases. However, from the results of a fundamental experiment,^17^ increasing the intraventricular concentration of VCM more than necessary was found not to cause any bacteriological effects and it is undesirable in terms of adverse effects. That is, it is believed that there is high risk of intraventricular administration of VCM at 20 or 10 mg leading to overdose in newborns. In contrast, in the investigation carried out in this study, it was confirmed that sufficient concentration in the CSF is maintained in cases with a VCM intraventricular administration of 5 mg. Moreover, it was found that the CSF concentration may still be sufficiently maintained at 72 hours following administration. As a result, it was believed that sufficient management was possible with 5 mg as the initial intraventricular administration for newborns.

Regarding the average VCM concentration in the CSF in adults, some studies mention that the trough level of CSF was 16.5 (10.0–25.4) mg/L^22^ after the administration of 5 mg, with a median trough level of the CSF of 26.8 (5.0–236) mg/L.^14^

Furthermore, regarding the interval of intraventricular VCM administration, administration in 12- to 24-hour intervals is the typical current regimen. The VCM CSF concentration varies greatly depending on the administration dose; however, in this investigation, it was possible to maintain a trough of ≥10 mg/L at 72 hours following administration, even at a dose of 5 mg. Conversely, regarding the conventional administration interval, the possibility of the concentration in the CSF further increasing is of concern due to additional administration being carried out at high concentrations. However, it has been reported that the CSF concentration declines when there is an excessive amount of discarded CSF ^16^). From the fact that the result of VCM concentration CSF varies depending on the case, the possibility of the concentration significantly declining depending on the patient's condition cannot be ruled out. Therefore, the 5 mg dose is considered to be sufficient regarding VCM administration in newborns, and it is believed that chronologically monitoring the concentration in the CSF is required to determine the optimal administration interval. Therefore, the usefulness of monitoring has been reported in previous studies in the pertinent literature.^5,15,18^

There are some limitations associated with this study. First of all, the described tendencies could not be investigated by a statistical analysis because the number of cases was small. However, since such cases are rare and this study includes a large number of cases in comparison with previous studies, including low weight infants, these findings are thus considered to be valuable. The background factors varied, thus potentially affecting the results; however, due to the fact that the concentration in the CSF 24 hours following administration was maintained even with respect to cases with excessive CSF disposal, it was believed that a sufficient therapeutic effect may be maintained even when the conventional 20 mg administration is reduced to 5 mg.

From the above, it is believed that regarding the intraventricular administration of VCM in newborns, commencing with an administration dose of 5 mg and then determining the optimal interval by monitoring the CSF every 12 to 24 hours will lead to a good therapeutic effect as a result of maintaining an appropriate concentration and reducing the risk of adverse effects due to an excessive CSF concentration. Furthermore, the data for low birth weight infants, such as premature infants, etc., were also limited in this study, and it is believed that the further accumulation of cases is thus required in the future.

## CONCLUSION

An investigation was carried out to elucidate the optimal method for performing intraventricular VCM administration in newborns. Sufficient concentrations in the CSF were maintained at an intraventricular administration dose of 5 mg VCM, and the appropriate administration interval may be determined by monitoring the CSF concentration.
